# Impaired neural differentiation and glymphatic CSF flow in the *Ccdc39* rat model of neonatal hydrocephalus: genetic interaction with *L1cam*

**DOI:** 10.1242/dmm.040972

**Published:** 2019-11-21

**Authors:** A. Scott Emmert, Eri Iwasawa, Crystal Shula, Preston Schultz, Diana Lindquist, R. Scott Dunn, Elizabeth M. Fugate, Yueh-Chiang Hu, Francesco T. Mangano, June Goto

**Affiliations:** 1 Division of Pediatric Neurosurgery, Cincinnati Children's Hospital Medical Center, Cincinnati, OH 45229, USA; 2 Division of Radiology, Cincinnati Children's Hospital Medical Center, Cincinnati, OH 45229, USA; 3 Developmental Biology, Cincinnati Children's Hospital Medical Center, Cincinnati, OH 45229, USA

**Keywords:** Neonatal hydrocephalus, Genetic model, Neocorticogenesis, Glymphatic, CRISPR-Cas, Rat model

## Abstract

Neonatal hydrocephalus affects about one child per 1000 births and is a major congenital brain abnormality. We previously discovered a gene mutation within the coiled-coil domain-containing 39 (*Ccdc39*) gene, which causes the *progressive hydrocephalus* (*prh*) phenotype in mice due to lack of ependymal-cilia-mediated cerebrospinal fluid (CSF) flow. In this study, we used CRISPR/Cas9 to introduce the *Ccdc39* gene mutation into rats, which are more suitable for imaging and surgical experiments. The *Ccdc39^prh/prh^* mutants exhibited mild ventriculomegaly at postnatal day (P)5 that progressed into severe hydrocephalus by P11 (*P<*0.001). After P11, macrophage and neutrophil invasion along with subarachnoid hemorrhage were observed in mutant brains showing reduced neurofilament density, hypomyelination and increased cell death signals compared with wild-type brains. Significantly more macrophages entered the brain parenchyma at P5 before hemorrhaging was noted and increased expression of a pro-inflammatory factor (monocyte chemoattractant protein-1) was found in the cortical neural and endothelial cells in the mutant brains at P11. Glymphatic-mediated CSF circulation was progressively impaired along the middle cerebral artery from P11 as mutants developed severe hydrocephalus (*P*<0.001). In addition, *Ccdc39^prh/prh^* mutants with L1 cell adhesion molecule (*L1cam*) gene mutation, which causes X-linked human congenital hydrocephalus, showed an accelerated early hydrocephalus phenotype (*P*<0.05-0.01). Our findings in *Ccdc39^prh/prh^* mutant rats demonstrate a possible causal role of neuroinflammation in neonatal hydrocephalus development, which involves impaired cortical development and glymphatic CSF flow. Improved understanding of inflammatory responses and the glymphatic system in neonatal hydrocephalus could lead to new therapeutic strategies for this condition.

This article has an associated First Person interview with the joint first authors of the paper.

## Abstract (precis)

**Summary:** Glymphatic CSF circulation and development of the cerebral cortex are impaired in our new genetic rat model of neonatal hydrocephalus with the onset of parenchymal inflammation and hemorrhage.

## INTRODUCTION

Neonatal hydrocephalus is a devastating condition defined by the abnormal accumulation of cerebrospinal fluid (CSF) in the brain that may arise from both genetic and acquired causes and can lead to brain damage and neurocognitive and motor skill problems (Schrander-Stumpel and Fryns, 1998; Tully and Dobyns, 2014; Vinchon et al., 2012). Neonatal hydrocephalus affects approximately one child in every 1000 live births (Haverkamp et al., 1999; Schrander-Stumpel and Fryns, 1998; Stoll et al., 1992) and contains a genetic etiology in nearly 40% of cases (Haverkamp et al., 1999; Schrander-Stumpel and Fryns, 1998; Zhang et al., 2006). Recent advancements in human genetic approaches such as whole-exome sequencing in familial and consanguineous forms of congenital hydrocephalus have identified a limited number of genes related to the development of this disease in humans, including *L1CAM* (Rosenthal et al., 1992), *MPDZ* (Al-Dosari et al., 2013), *CCDC88C* (Ekici et al., 2010), *EML1* (Shaheen et al., 2017), *WDR81* (Shaheen et al., 2017), *TRIM71* (Furey et al., 2018a), *SMARCC1* (Furey et al., 2018a) and *PTCH1* (Furey et al., 2018a). Collectively, mutations in these genes account for approximately 15% of all congenital hydrocephalus cases (Furey et al., 2018b). However, the cellular events that are essential for neonatal hydrocephalus development and its interactions with the surrounding physiological networks of CSF circulation and absorption are poorly understood. For example, the role of the recently discovered glymphatic system, a brain-wide CSF and soluble compounds distribution system, in neonatal hydrocephalus is unknown. Defined as the glial-associated lymphatic system (glymphatic system) for its dependence on astrocytic aquaporin-4 (AQP4) channels and lymphatic-like role in the brain, the glymphatic system of perivascular spaces, which are lined by astrocytic foot processes with AQP4 channels and endothelial abluminal membranes, drains the brain of interstitial fluid and waste products into extracranial lymphatics (Iliff et al., 2012). Associated with the meningeal lymphatic network (Aspelund et al., 2015; Louveau et al., 2015), the glymphatic system is an area of promising research for its role in draining the CSF contents in healthy brains as well as those affected by neurological disorders such as Alzheimer's disease (Iliff et al., 2012), traumatic brain injury (Iliff et al., 2014) and hydrocephalus (Ringstad et al., 2017).

A genetic model of neonatal hydrocephalus involving the coiled-coil domain-containing 39 (*Ccdc39*) gene may help elucidate cellular mechanisms leading to the abnormal accumulation of cerebrospinal fluid in neonatal hydrocephalus, as this model shows a robust and 100% penetrant hydrocephalus phenotype in mice, which is not common in other rodent models. *Ccdc39* is selectively expressed in embryonic choroid plexus and ependymal cells on the medial wall of the ventricular forebrain (Abdelhamed et al., 2018), and the protein is localized to the axoneme of motile cilia (Merveille et al., 2011). In mice, *Ccdc39* gene mutation leads to ependymal cells with shorter cilia, with microtubules lacking the axonemal inner arm dynein, resulting in impaired ependymal cilia beating and intraventricular CSF flow (Abdelhamed et al., 2018). Although substantial strides have been made in characterizing the mechanisms of ciliary dysfunction (Lee, 2013) and CSF flow abnormalities (Date et al., 2019; Olstad et al., 2019) caused by ciliary gene mutations, the pathophysiologic downstream processes whereby impaired CSF flow leads to hydrocephalus are still unsolved. The small size of murine models inhibits the use of surgical procedures conducive to studying these processes at early developmental time points; however, such procedures could be performed on the brains of larger mammalian models of neonatal hydrocephalus generated using the CRISPR/Cas9 genome editing system.

The emergence of CRISPR/Cas9 technology provides an accessible method for generating transgenic rat models of congenital hydrocephalus (Emmert et al., 2019) that were unfeasible with previous genetic techniques (Mashimo, 2014; Sander and Joung, 2014). Furthermore, CRISPR/Cas9 offers the opportunity to test genetic modifiers and possible genetic interactions that determine disease severity in congenital hydrocephalus. For instance, X-linked hydrocephalus (XLH), which can result from mutations in the L1 cell adhesion molecule (*L1cam*) gene in mice (Dahme et al., 1997; Demyanenko et al., 1999), rats (Emmert et al., 2019) and humans (Adle-Biassette et al., 2013; Dahme et al., 1997; Rosenthal et al., 1992), varies in severity from hydrocephalus with multiple structural abnormalities and prenatal death to a milder phenotype with cognitive impairment or isolated symptoms even within the same family (Fryns et al., 1991; Serville et al., 1992). CRISPR/Cas9-generated rodent models of congenital hydrocephalus resulting from mutations in different hydrocephalus-related genes, such as *L1cam* and *Ccdc39*, can be interbred to investigate epistatic interactions previously believed to affect mutation penetrance and ventricular size in other models of hydrocephalus (Weller and Gärtner, 2001; Zhang et al., 2006).

Inflammation related to neonatal hydrocephalus has been investigated primarily in posthemorrhagic hydrocephalus both in animal models (Gram et al., 2014; Yung et al., 2011) and in patients (Heep et al., 2004; Klebe et al., 2019; Sävman et al., 2002). These studies show an increase in several cytokines in the CSF, which eventually causes oxidative stress and further damages the brain tissue. Although little is known about the inflammatory response in cases of neonatal hydrocephalus without preceding hemorrhage, inflammation has been shown to be causal in hydrocephalus models (Abdi et al., 2018; Botfield et al., 2013; Lattke et al., 2012). Periventricular white matter has been reported as a specifically vulnerable region to the hydrocephalus insult in neonates both in human and animal models of hydrocephalus (Del Bigio et al., 2003; Hanlo et al., 1997), possibly because of the direct physical stress from periventricular distention or indirectly by hypoxic-ischemic stress (du Plessis, 1998) or other mechanisms, including inflammation.

Using CRISPR/Cas9 to model neonatal hydrocephalus in rats, we generated a *Ccdc39* knockout line in Sprague Dawley rats. Here, we studied the genetic interaction of two hydrocephalus-related genes, *L1cam* and *Ccdc39*, through genetic, survival and growth characterization of the *Ccdc39^prh/prh^* mutant rat in the presence and absence of an *L1cam*-null allele. We also examined spatiotemporal inflammatory reactions along with cortical development in this novel neonatal hydrocephalus model. To conclude, we investigated the pattern of CSF circulation through the glymphatic system for the first time in neonatal hydrocephalus, using Evans Blue dye injected into the cisterna magna of control and *Ccdc39^prh/prh^* mutant rats.

## RESULTS

### CRISPR/Cas9-mediated modeling of the *prh* mutation (*Ccdc39^c.916+2T>A^*) in rats

**Fig. 1.**
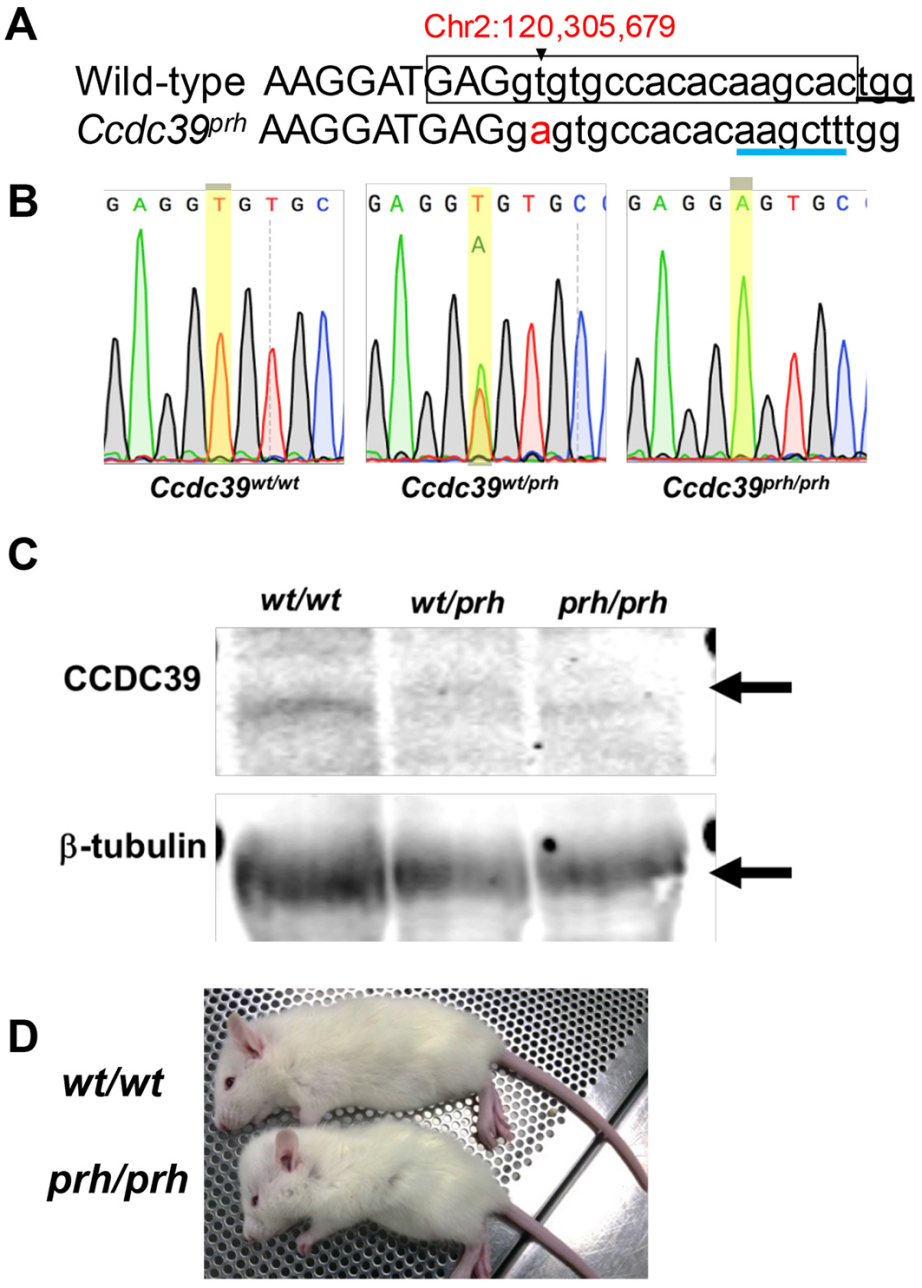
**CRISPR/Cas9-mediated modeling of the *prh* mutation (*Ccdc39^c.916+2T>A^*) in rats.** (A) Wild-type and *prh* (*Ccdc39^prh^*) DNA sequence harboring the critical thymine nucleotide (black arrowhead) required for proper splicing of the *Ccdc39* gene in rats (chr2:g.120305679). Capital letters, exon; boxed letters, guide RNA sequence; black underline, protospacer adjacent motif; blue underline, engineered *Hin*dIII restriction site; red, T>A mutation. (B) Genomic DNA Sanger sequencing traces of wild-type (*Ccdc39^wt/wt^*), heterozygous (*Ccdc39^wt/prh^*) and mutant (*Ccdc39^prh/prh^*) rat offspring generated with CRISPR/Cas9 genome editing. *Ccdc39^prh/prh^* rats exhibit the intended homozygous (*Ccdc39^c.916+2T>A^*) change. (C) Immunoblot analysis on P11 rat brain lysate confirms the loss of CCDC39 in the homozygous mutant. CCDC39 protein expression (approximately 110 kDa; black arrow) was reduced to approximately 50% and 0% of the normal expression level in heterozygous and homozygous mutant rats, respectively. β-tubulin was the loading control. (D) Gross picture of wild-type (*Ccdc39^wt/wt^*) and mutant (*Ccdc39^prh/prh^*) rats show the dome-shaped head of a mutant rat at P25.

We previously identified a *Ccdc39^prh^* mutation (Abdelhamed et al., 2018) in the *progressive hydrocephalus* (*prh*) mouse mutant with neonatal hydrocephalus (Stottmann et al., 2011). To efficiently and specifically induce the same mutation in rats, CRISPR guide RNA (gRNA), primers and oligonucleotide donor repair templates were designed to introduce the homozygous chr2:g.120305679A>T change that creates a splice site (*Ccdc39^c.916+2T^*) mutation in the rat *Ccdc39* gene (Fig. 1A, Table 1). Guide RNA sequences were selected based upon favorable on-target and off-target scores according to CRISPR guide design tools Benchling version 1 (https://benchling.com/academic) and CRISPOR (http://crispor.tefor.net/). Of the rats born from CRISPR-modified embryos (*n*=26), two rats exhibited the intended *Ccdc39^c.916+2T>A^* mutation upon Sanger sequencing, whereas other edited offspring demonstrated insertions and deletions (*n*=10) around the targeted site. F0 animals with mosaicism were bred to wild-type Sprague Dawley rats to generate F1 heterozygous rats (*Ccdc39^wt/prh^*), which show the *Ccdc39^c.916+2T>A^* change with an adenine peak of approximately half the intensity of the wild-type thymine peak in the sequencing chromatogram (Fig. 1B). The F1 *Ccdc39^wt/prh^* heterozygous rats were subsequently bred to generate homozygous *Ccdc39^prh/prh^* rat mutants (*Ccdc39^prh/prh^*). The homozygous mutation was confirmed by Sanger sequencing of F1-F2 pups. Subsequent generations were genotyped with TaqMan probes detecting the difference between T and A at *Ccdc39^c.916+2T^* (see Materials and Methods). In western blotting, CCDC39 protein expression (approximately 110 kDa; black arrow in Fig. 1C) was significantly reduced in *Ccdc39^wt/prh^* (*n*=2) rats and completely eliminated in *Ccdc39^prh/prh^* (*n*=2) rats relative to wild-type littermates (*n*=2) (Fig. 1C), which aligns with our previous finding that the *prh* mouse mutant exhibits loss of CCDC39 protein as a result of abnormal mRNA splicing (Abdelhamed et al., 2018).

**Table 1.**
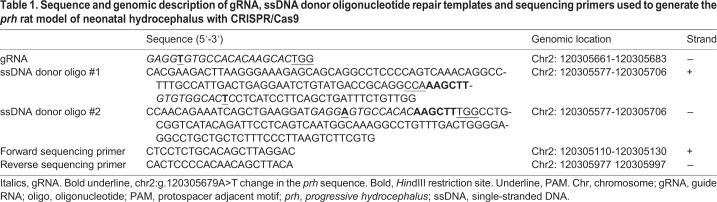
**Sequence and genomic description of gRNA, ssDNA donor oligonucleotide repair templates and sequencing primers used to generate the *prh* rat model of neonatal hydrocephalus with CRISPR/Cas9**

### Progressive hydrocephalus in *Ccdc39^prh/prh^* mutant rats

**Fig. 2.**
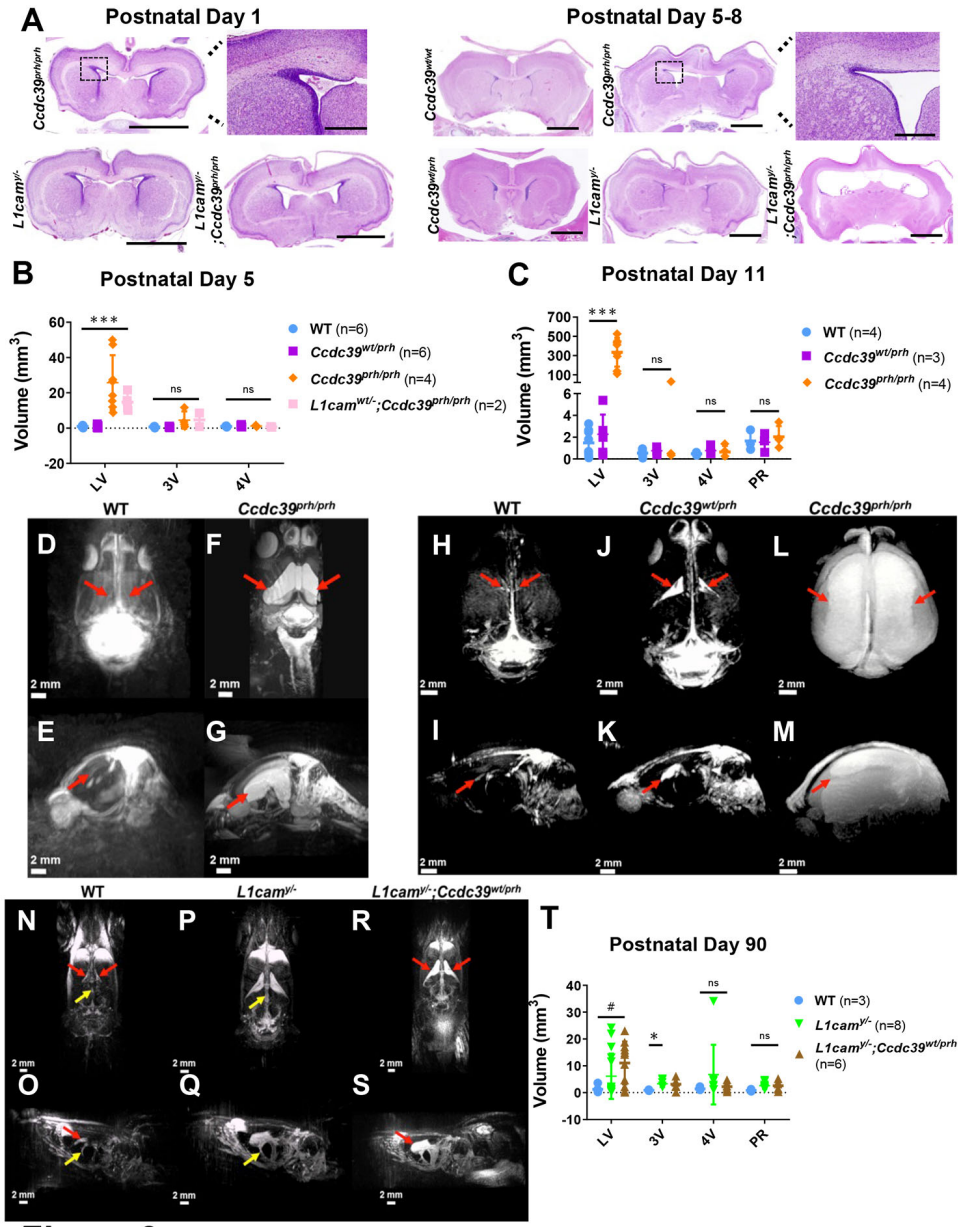
**Postnatal hydrocephalus in *Ccdc39* mutant rats and genetic interaction with the *L1cam*-null allele.** (A) H&E staining of P1 and P5-P8 wild-type (*Ccdc39^wt/wt^*) (*n*=5), *Ccdc39* heterozygous (*Ccdc39^wt/prh^*) (*n*=2), *Ccdc39* homozygous mutant (*Ccdc39^prh/prh^*) (*n*=6 at P1, *n*=4 at P5-8), *L1cam* mutant (*L1cam^y/−^*) (*n*=5 at P1, *n*=2 at P5-8) and *L1cam;Ccdc39* double-mutant (*L1cam^y/−^;Ccdc39^prh/prh^*) rats (*n*=4 at P1, *n*=3 at P5-8). Ventricular dilation was observed in P1 *L1cam^y/−^;Ccdc39^prh/prh^* double mutants and P5 *Ccdc39^prh/prh^* mutants, with the most severe dilation observed in P5 *L1cam^y/−^;Ccdc39^prh/prh^* double mutants. Each area of the dashed square overlaying P1 and P5 *Ccdc39^prh/prh^* mutants is shown with a higher magnification image. (B,C) Volumetric analysis of the lateral ventricles (LV), third ventricle (3V), fourth ventricle (4V) and pineal recess (PR) shows that *Ccdc39^prh/prh^* (*n*=4) and *L1cam^wt/−^;Ccdc39^prh/prh^* (*n*=2) mutants demonstrate significantly dilated lateral ventricles beginning at P5, which develops into severe hydrocephalus at P11 relative to *Ccdc39^wt/prh^* (*n*=3) and wild-type (*n*=4) rats. ***P<0.001; ns, not significant determined by two-way ANOVA and Holm–Sidak's multiple comparisons test. (D-S) Representative three-dimensional T2-weighted MR images of wild-type (D,E,H,I,N,O), *Ccdc39^prh/prh^* (F,G,L,M), *Ccdc39^wt/prh^* (J,K), *L1cam^y/−^* (P,Q) and *L1cam^y/−^;Ccdc39^wt/prh^* (R,S) rats at P5 (D-G), P11 (H-M) and P90 (N-S). Red arrows indicate lateral ventricles. Yellow arrows indicate third ventricle. (T) *L1cam^y/−^* (*n*=8) and *L1cam^y/−^;Ccdc39^wt/prh^* (*n*=6) rats demonstrate a similarly enlarged third ventricle and lateral ventricles at P90. Results are presented as individual replicates overlaying the mean±s.d. *^#^P<*0.05 between wild type and *L1cam^y/−^;Ccdc39^wt/prh^* determined as for B,C. *P<0.05 between wild type and *L1cam^y/−^* determined as for B,C. Scale bars: 2 mm (A, D-S); 500 μm (magnified images in A).

**Fig. 3.**
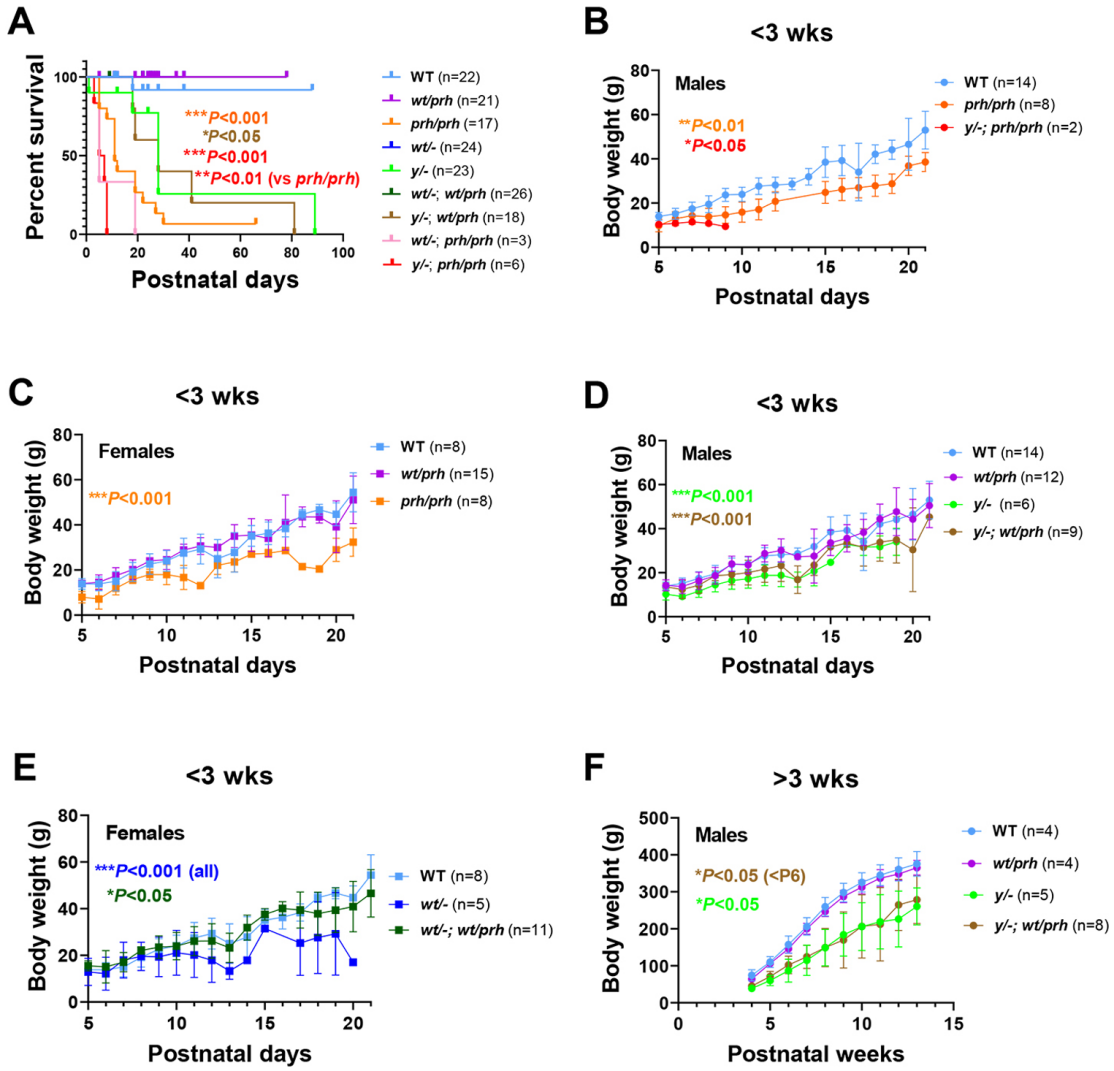
**Reduced survival and growth in *Ccdc39* mutant rats with the *L1cam*-null allele.** (A) Survival rate of male and female postnatal wild-type (WT; light blue), *Ccdc39* heterozygous (*wt/prh*; purple), *Ccdc39* mutant (*prh/prh*; orange), *L1cam* heterozygous (*wt/−*; dark blue), *L1cam* mutant (*y/−*; lime green), *L1cam* heterozygous*;Ccdc39* heterozygous (*wt*/−; *wt/prh*; forest green), *L1cam* mutant*;Ccdc39* heterozygous (*y/−*; *wt/prh*; brown), *L1cam* heterozygous*;Ccdc39* mutant (*wt/−*; *prh/prh*; pink) and *L1cam;Ccdc39* double-mutant (*y/−*; *prh/prh*; red) rats reveals that *Ccdc39* mutant (*n*=17) and *L1cam* mutant*;Ccdc39* heterozygous (*n*=18) rats recapitulate the pre-weaning phase mortality of *Ccdc39* mutant mice. The survival phenotype in *Ccdc39* mutant rats is exaggerated when this model is crossed with the *L1cam*-null allele, as shown by *L1cam;Ccdc39* double mutants (*n*=6) (*P=*0.0013). (B-F) Body weight analysis of male (B,D,F; circles) and female (C,E; squares) postnatal wild-type, *Ccdc39* mutant, double-mutant, *Ccdc39* heterozygous, *L1cam* mutant, *L1cam* mutant*;Ccdc39* heterozygous, *L1cam* heterozygous and double-heterozygous rats younger (B-E) and older (F) than 3 weeks (wks) of age demonstrates that *Ccdc39* mutants (*n*=8) exhibit mild growth delays throughout the first 3 weeks of life, which are exaggerated by the presence of a *L1cam*-null allele in *L1cam;Ccdc39* double mutants (*n*=2). *L1cam* heterozygous (*n*=5) and *L1cam* heterozygous*;Ccdc39* heterozygous (*n*=11) rats exhibit similar reductions in growth over 3 weeks (E). *L1cam* mutant rats (*n*=5-6) similarly exhibit decreased body weight from birth through 3 months of age, including when harboring a *Ccdc39*-null allele, as shown by *L1cam* mutant*;Ccdc39* heterozygous rats (*n*=8) (F). Male and female rats were analyzed separately for body weight due to sex differences in feeding behavior in rats (Fukushima et al., 2015). Results are presented as mean±s.d. *P*-values, which were determined by log-rank test (survival) or nonlinear regression (body weight) between each genotype and the wild type (unless otherwise noted) are indicated on the graph and color-coded by genotype. Allele abbreviations and color coding for each genotype presented in B-F are the same as for A.

*Ccdc39^prh/prh^* rats exhibited dome-shaped heads (Fig. 1D) and developed progressive postnatal hydrocephalus (Fig. 2A-M) over 2 weeks. Male and female *Ccdc39^prh/prh^* mutant pups demonstrated gradual growth delays (*n^wt/wt^=*8-14, *n^prh/prh^=*8; *P<*0.01-0.001) and early mortality in life (*n^wt/wt^=*22, *n^prh/prh^=*17; *P<*0.001) (Fig. 3A-C). In histology, P5-P8, but not P1, *Ccdc39^prh/prh^* rats exhibited dilation of the lateral ventricles, smaller subventricular zone and thinning of the cortical mantle relative to wild-type and heterozygous littermates (Fig. 2A). Three-dimensional (3D) volumetric T2-weighted MR images were acquired through the brains of wild-type and *Ccdc39^prh/prh^* rats. Volumetric analysis of the lateral ventricles, third ventricle, fourth ventricle and pineal recess revealed that *Ccdc39^prh/prh^* rats demonstrate mild enlargement of the lateral ventricles beginning at P5 (Fig. 2A,B,F,G) that progressed into severe ventriculomegaly relative to control littermates by P11 (*n^wt/wt^=*4-6, *n^wt/prh^=*3-6, *n^prh/prh^=*4; *P<*0.001) (Fig. 2C,L,M). However, no differences were found in the volumes of the third ventricle, fourth ventricle or pineal recess in *Ccdc39^prh/prh^* (*n*=4) and *L1cam* heterozygous/*Ccdc39* mutant (*L1cam^wt/−^;Ccdc39^prh/prh^*, *n*=2) rats relative to control (*n*=4-6) and heterozygous (*n*=3-6) littermates at either P5 or P11.

### Interaction between hydrocephalus-causing *Ccdc39* and *L1cam* gene mutations in *L1cam^y/−^;Ccdc39^prh/prh^* double-mutant rats worsens the survival, growth and ventriculomegaly of *Ccdc39^prh/prh^* mutant rats

To investigate the epistatic interactions between known hydrocephalus-causing gene mutations, we crossed the *Ccdc39^prh^* allele with the *L1cam^−^* allele that we generated recently (Emmert et al., 2019). *L1cam^y/−^* single mutant rats show delayed growth and develop mild enlargement of the fourth and lateral ventricles by 3 and 6 weeks postnatally, respectively (Emmert et al., 2019), modeling a mild form of XLH. Survival rate and body weight analyses of male and female postnatal wild-type, *L1cam* heterozygous/*Ccdc39* heterozygous (*L1cam^wt/−^;Ccdc39^wt/prh^*), *L1cam* mutant/*Ccdc39* heterozygous (*L1cam^y/−^;Ccdc39^wt/prh^*), double-mutant (*L1cam^y/−^;Ccdc39^prh/prh^*), and *L1cam* heterozygous/*Ccdc39* mutant (*L1cam^wt/−^;Ccdc39^prh/prh^*) rats revealed that the stunted growth and premature mortality of *Ccdc39^prh/prh^* rats were exaggerated in the presence of the *L1cam*-null allele, as evidenced by the decreased survival and growth of male *L1cam^y/−^;Ccdc39^prh/prh^* rats (*n*=6 and *n*=2, respectively; *P<*0.001 between wild-type and *L1cam^y/−^;Ccdc39^prh/prh^* and *P<*0.01 between *Ccdc39^prh/prh^* and *L1cam^y/−^;Ccdc39^prh/prh^* for survival; *P<*0.05 between *Ccdc39^prh/prh^* and *L1cam^y/−^;Ccdc39^prh/prh^* for body weight) (Fig. 3A,B). Furthermore, *L1cam^y/−^;Ccdc39^prh/prh^* double mutants exhibited mild dilation of the lateral ventricles as early as P1 in histology (*n*=4; Fig. 2A), which was not seen in single mutants. These data suggest that mutations in *L1cam* and *Ccdc39* could converge phenotypically in hydrocephalus and that mutation in both genes accelerates the development of neonatal hydrocephalus (Fig. 2A). However, the heterozygous loss of the *Ccdc39* allele did not worsen the survival (Fig. 3A) or growth phenotype (Fig. 3D,F) of *L1cam^y/−^* (*n*=23 for survival, *n*=5-6 for weight) in *L1cam^y/−^;Ccdc39^wt/prh^* rats (*n*=18 for survival, *n*=9 for weight) either in the early postnatal period or through 3 months of age. Also, heterozygous loss of *L1cam^wt/−^* did not affect the early lethality of *Ccdc39^prh/prh^* single mutants (*L1cam^wt/−^;Ccdc39^prh/prh^ n*=3; Fig. 3A,). Similarly, *L1cam^wt/−^;Ccdc39^wt/prh^* rats (*n*=11) demonstrated moderate decreases in growth compared with wild-type animals (*n*=8; *P<*0.05) but did not differ in weight from *L1cam^wt/−^* rats (*n*=5) (Fig. 3E). Additionally, *L1cam^wt/−^;Ccdc39^wt/prh^* rats (*n*=26) did not exhibit the hydrocephalus (Fig. 2A) or early lethality (Fig. 3A) of *Ccdc39^prh/prh^* or *L1cam^y/−^;Ccdc39^prh/prh^* rats.

We further examined the genetic interaction between *L1cam* and *Ccdc39* gene mutations in brain development by performing 3D volumetry on T2-weighted magnetic resonance imaging. At P5, *L1cam^wt/−^;Ccdc39^prh/prh^* rats (*n*=2) exhibited mild hydrocephalus relative to wild-type littermates (*n*=6, *P<*0.001) but did not differ in the volume of their lateral ventricles compared with *Ccdc39^prh/prh^* rats (*n*=4) (Fig. 2B,D-G). At P90, *L1cam^y/−^;Ccdc39^wt/prh^* rats (*n*=6) exhibited dilation of the lateral ventricles relative to wild types (*n*=3, *P<*0.05) and a similar level of ventriculomegaly to that of the *L1cam^y/−^* rat (*n*=8) (Fig. 2N-T). Magnetic resonance imaging (MRI) of *L1cam^y/−^;Ccdc39^prh/prh^* rats was difficult to acquire because of their early lethality.

### Inflammation and subarachnoid hemorrhage are seen in *Ccdc39^prh/prh^* mutant rats

**Fig. 4.**
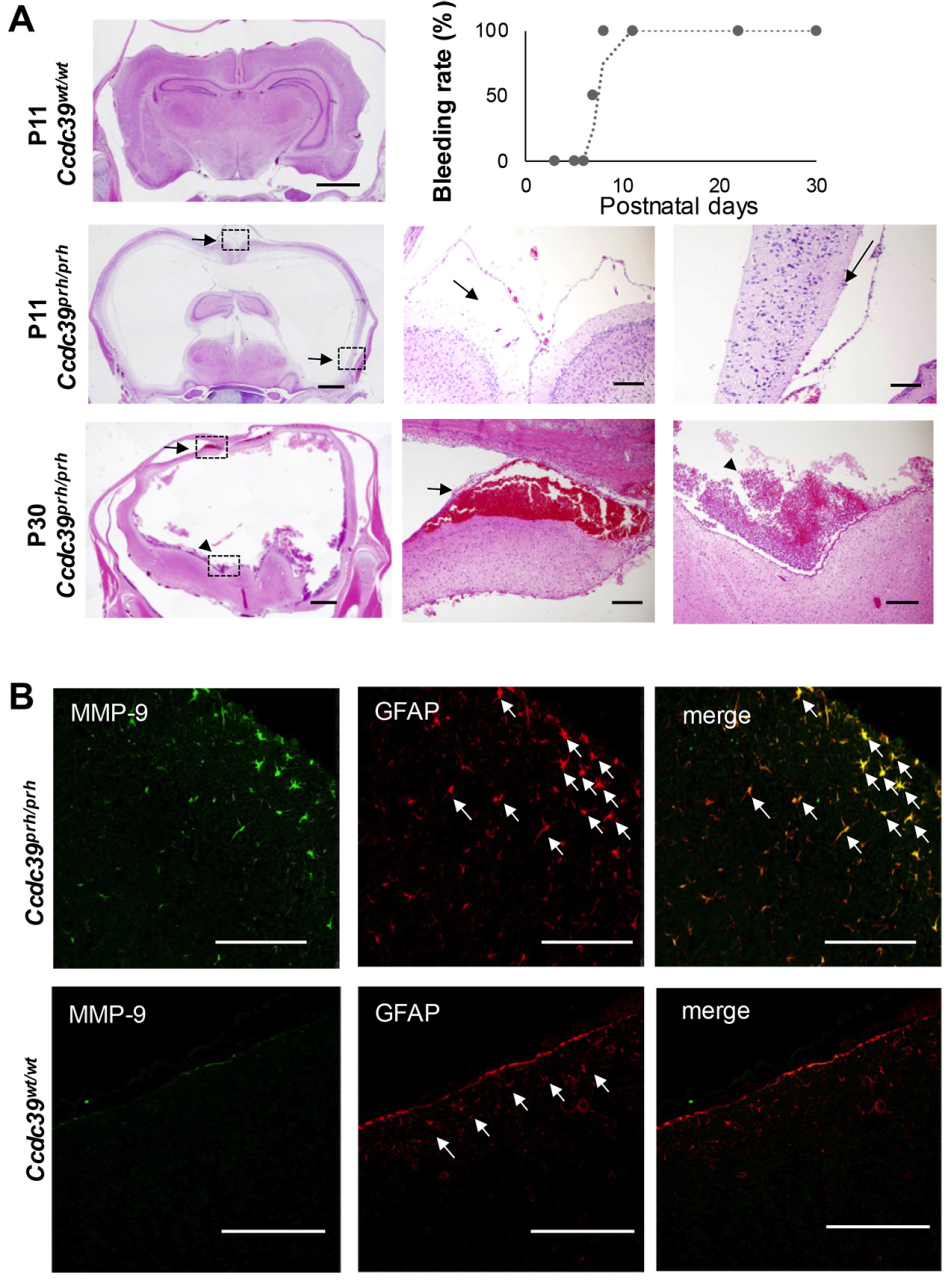
**Bleeding in the *Ccdc39^prh/prh^* mutant rat model of neonatal hydrocephalus.** (A) The *Ccdc39* homozygous mutant (*Ccdc39^prh/prh^*) rat shows subtle subarachnoid bleeding along with arachnoid membrane dissociation from brain parenchyma (arrows) starting from P9, and the rate of bleeding reaches 100% by P11. The hemorrhage is found in both subarachnoid/subpial area (arrows) and lateral ventricles (arrowheads) at P30 (graph of bleeding rate; *n*=16 of *Ccdc39* mutants, P1-P30). (B) The activated form of MMP9 staining at P11 shows MMP9 activation near the brain surface containing the pia-dissociated area in *Ccdc39* homozygous mutant (*Ccdc39^prh/prh^*) rats and is expressed in GFAP-positive astrocytes (white arrows) (*n*=1 in each group). Scale bars: 2 mm (A, left column); 200 μm (A, middle and right columns); 100 μm (B).

**Fig. 5.**
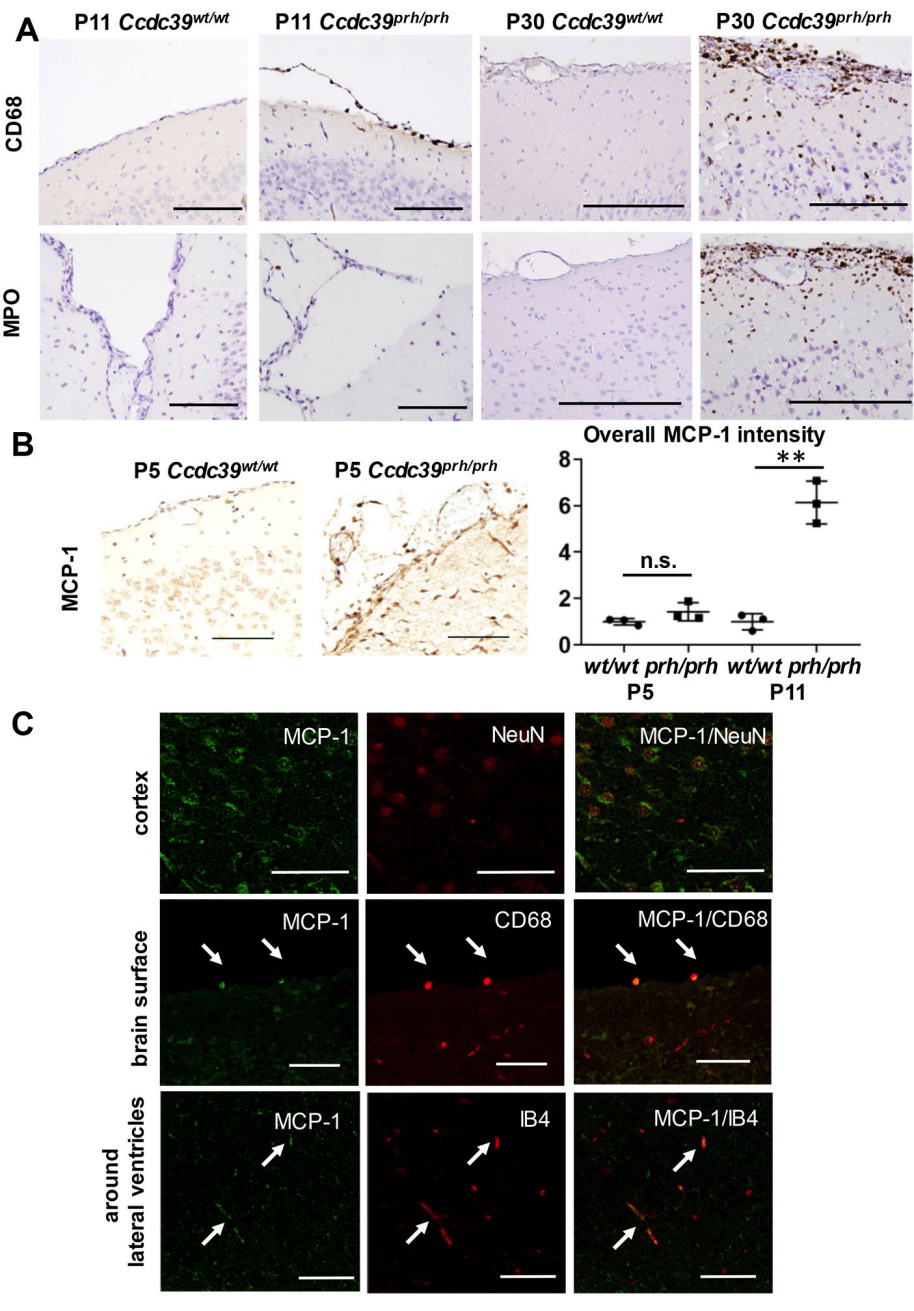
**Neuroinflammation emerges during the early stage of ventriculomegaly in *Ccdc39* mutant rats.** (A) CD68-positive macrophages migrate to the subarachnoid space at P11 (*n*=2) and invade into parenchyma at P30 (*n*=1) in *Ccdc39* homozygous mutants (*Ccdc39^prh/prh^*). MPO-positive neutrophils are barely seen in wild types and *Ccdc39^prh/prh^* mutants at P11 (*n*=2, in each group), but distinctly accumulate over the brain surface with the parenchymal invasion at P30 in *Ccdc39^prh/prh^* mutant rats (*n*=1, in each group). (B) Greater signal of MCP1 expression was seen beginning at P5 in *Ccdc39^prh/prh^* mutant rats, which reached statistical significance at P11 (mean±s.d., *P*=0.14 for P5, **P<0.01 for P11; *n*=3 in each group). (C) MCP1 signals are seen in NeuN-positive neurons, CD68-positive macrophages and endothelial cells labeled with isolectin B4 (IB4) in *Ccdc39^prh/prh^* mutants at P11 (*n*=3). Scale bars: 200 μm (A,B); 100 μm (C, top and middle rows); 50 μm (C, bottom row).

To describe the molecular events of hydrocephalus in *Ccdc39^prh/prh^* mutant rats, we first characterized the developmental time course of intracranial hemorrhage in serial hematoxylin and eosin (H&E) staining of the postnatal *Ccdc39^prh/prh^* mutant rats. A small amount of bleeding was seen in the subarachnoid area of P8 *Ccdc39^prh/prh^* mutant rats and became massive in both the subarachnoid space and subpial area, and later into the lateral ventricles by P30 (Fig. 4A). No bleeding was seen in wild-type rats by P30 (*n^prh/prh^*=16, *n^wt/wt^*=8). Some amount of hemorrhage was located under pia mater. Because either pia mater or subarachnoid membrane seemed to be dissociated from the brain surface along with fluid accumulation in the space between the membrane and brain parenchyma (arrows in Fig. 4A), which was not observed wild type rats, we investigated possible cellular processes that might cause pial/arachnoid membrane dissociation from the brain surface and eventual bleeding. Staining for the activated form of matrix metalloproteinase 9 (MMP9) showed its upregulation near the brain surface of *Ccdc39^prh/prh^* mutant rats at P11, and it was expressed in glial fibrillary acidic protein (GFAP)-positive astrocytes (Fig. 4B). We hypothesized that this abnormally activated MMP9 and possibly the eventual bleeding occurred secondarily to the inflammatory reaction in hydrocephalus. When assessing inflammatory reactions in *Ccdc39^prh/prh^* mutant rats, significantly more CD68-positive activated macrophages first appeared in the periventricular white matter and striatum at P5 compared with wild-type rats (*n*=3 in each group, *P*=0.046; Fig. S1A) and were subsequently seen in the subarachnoid area at P11. Myeloperoxidase (MPO)-positive neutrophils filled the subarachnoid space and partly filled the perivascular space at P30 (Fig. 5A). This inflammatory cell invasion into the perivascular space was not seen prior to P30. Interestingly, there was more signal of a candidate factor of inflammatory cell migration, monocyte chemoattractant protein 1 (MCP1), as early as P5 in *Ccdc39^prh/prh^* rats, although not statistically significant (*n*=3 in each group, *P*=0.14; Fig. 5B). The upregulation of MCP1 expression was statistically significant at P11 (*n*=3 in each group, *P*<0.01; Fig. 5B) and was mostly seen in neuronal nuclei (NeuN)-positive neurons, CD68-positive macrophages and endothelial cells labeled with IB4 (Fig. 5C; Fig. S1B).

### Impaired neural differentiation and increased cell death in *Ccdc39^prh/prh^* rats

**Fig. 6.**
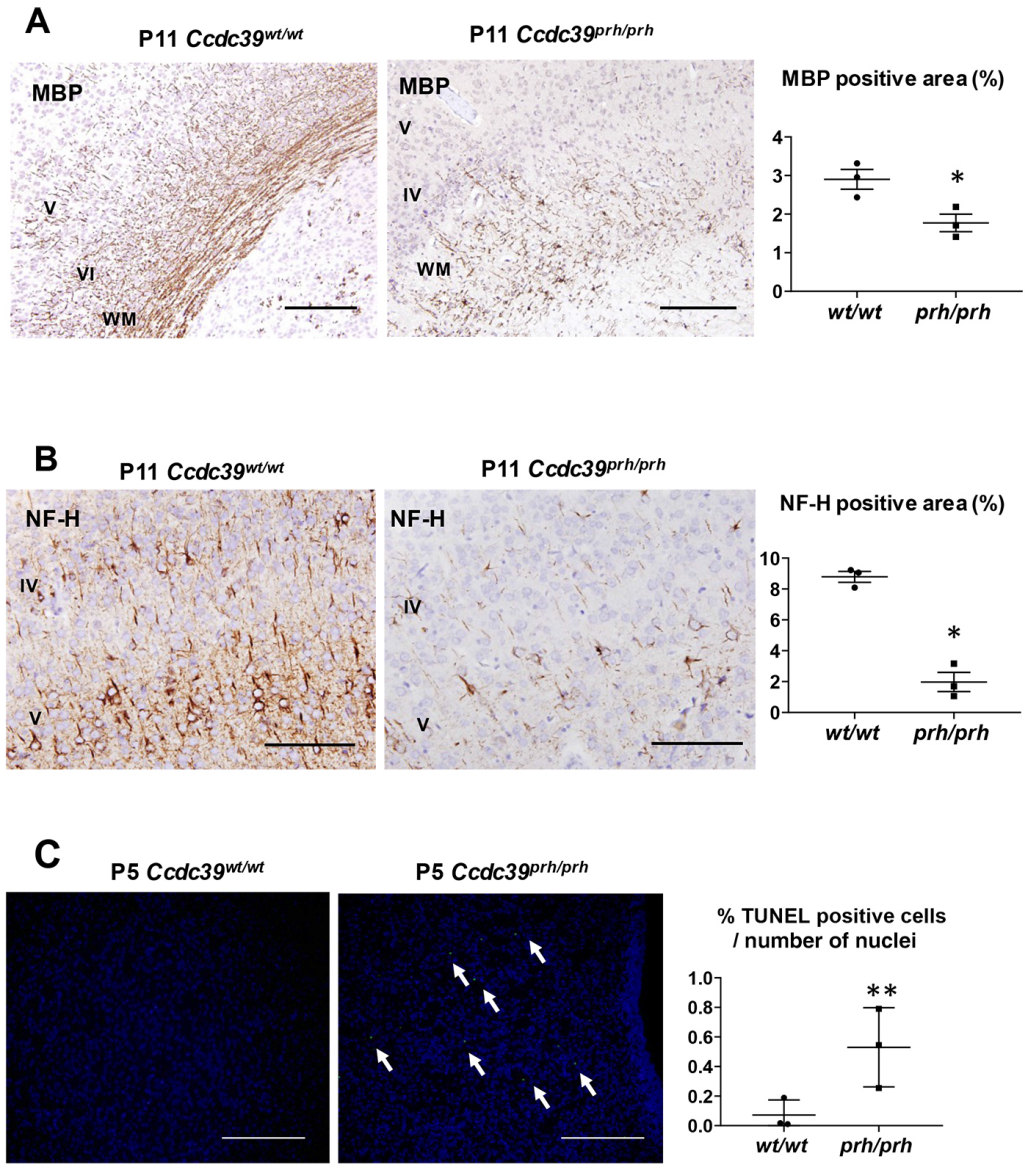
**Myelination and neurofilament development are impaired and cell death signals are increased in *Ccdc39^prh/prh^* mutant brains.** (A) MBP staining shows significantly reduced myelination in the brain cortex in the rat *Ccdc39* homozygous mutant (*Ccdc39^prh/prh^*) compared with wild-type (*Ccdc39^wt/wt^*) rats at P11 (mean±s.e.m., *n*=3 in each group). (B) NF-H staining shows significantly reduced neurofilament densities in *Ccdc39* homozygous mutant (*Ccdc39^prh/prh^*) compared with wild-type (*Ccdc39^wt/wt^*) rats at P11 (mean±s.e.m., *n*=3 in each group). (C) In *Ccdc39* homozygous mutant (*Ccdc39^prh/prh^*) rats, compared with wild type, more TUNEL-positive cells (arrows) are seen in the periventricular region at P1-P5 (mean±s.e.m., *n*=3 in each group). Scale bars: 100 μm (A); 200 μm (B,C). *P<0.05, **P<0.01. IV, cortical layer 4; V, cortical layer 5; VI, cortical layer 6; WM, white matter.

To assess the development of the cerebral cortices, we evaluated myelin basic protein (MBP) staining in cerebral white matter. The MBP-positive area was significantly reduced in *Ccdc39^prh/prh^* mutant rats compared with *Ccdc39^wt/wt^* wild-type rats at P11 (Fig. 6A). At P5, myelination had barely started, even in *Ccdc39^wt/wt^* wild-type rats (Fig. S2A), and there was no significant difference in the number of MBP-positive mature oligodendrocytes between *Ccdc39^prh/prh^* mutant and *Ccdc39^wt/wt^* wild-type rats (*n*=3 in each group, *P*=0.18; Fig. S2A). The impairment in myelin formation at P11 was accompanied by impaired neurofilament expression, as assessed with neurofilament-H (NF-H) subunit staining, which showed sparse and shortened neurofilaments in *Ccdc39^prh/prh^* mutant rats at P11 (Fig. 6B). There was a tendency for reduced neurofilament formation in *Ccdc39^prh/prh^* mutant rats at P5, although this phenomenon was not statistically significant (*n*=3 in each group, *P*=0.11; Fig. S2B). We next evaluated cell death signals using TUNEL staining in *Ccdc39^prh/prh^* mutant rats at P1-P5. Increased cell death, as measured by TUNEL-positive cells, was detected around the periventricular region in *Ccdc39^prh/prh^* mutant rat brains compared with wild-type rats (Fig. 6C).

### Impaired CSF flow through the glymphatic system in the *Ccdc39^prh/prh^* mutant rat model of neonatal hydrocephalus

**Fig. 7.**
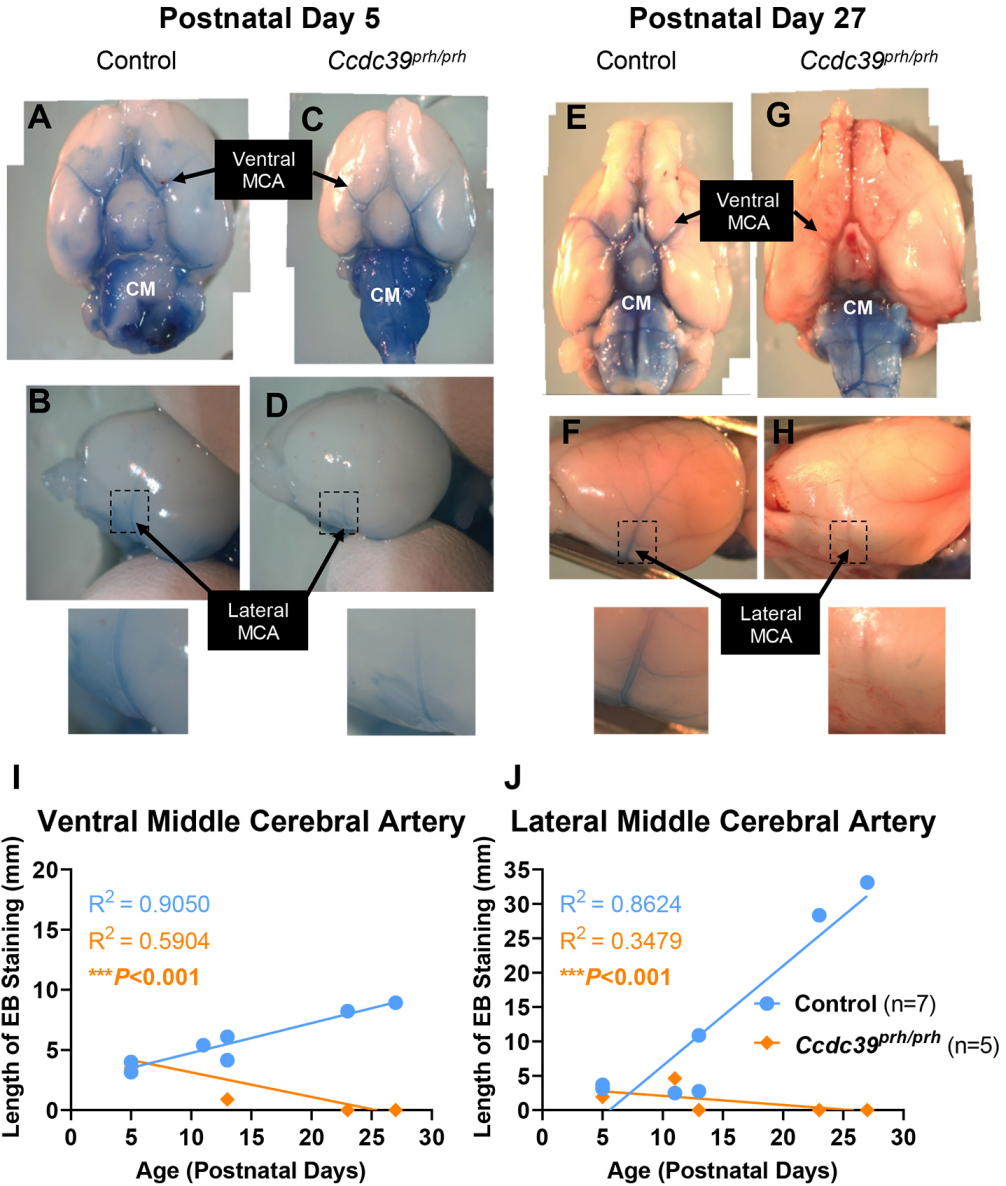
**Cerebrospinal fluid circulation through the glymphatic system is impaired in *Ccdc39^prh/prh^* mutants.** (A-F) Representative images of P5 control (A,B) and mutant (*Ccdc39^prh/prh^*) (C,D) rat brains and P27 control (E,F) and mutant (G,H) rat brains injected with Evans Blue dye into the cisterna magna to trace the circulation of CSF through the glymphatic system. (I,J) Quantification of the length of EB staining along the ventral (I) and lateral (J) surfaces of the middle cerebral artery in control (*n*=7) and mutant (*n*=5) rats. Results are presented as individual replicates. The legend in J also applies to I. ***P<0.001 determined by comparing fits of nonlinear regression curves and best-fit values. CM, cisterna magna; EB, Evans Blue; MCA, middle cerebral artery.

We investigated the CSF circulation path within the glymphatic system at different stages of hydrocephalus in the *Ccdc39^prh/prh^* mutant rat model by injecting Evans Blue dye into the cisterna magna of mutants (*n*=7) ranging in age from P5 to P27 and age-matched controls (*n*=5). Evans Blue is a low molecular weight tracing dye with a high affinity for serum albumin that accumulates in perivascular spaces, such as the Virchow–Robin spaces of the glymphatic system, after intracerebral injection (Maloveska et al., 2018; Stoelinga and van Munster, 1967). It has been used as a marker for blood–brain barrier integrity, meningeal lymphatic vasculature and glymphatic flux because of its colorimetric, fluorescent and protein-binding qualities (Maloveska et al., 2018; Stoelinga and van Munster, 1967; Wolf et al., 2019). Likewise, various groups have used fluorescently labeled protein tracers injected into the cisterna magna to show flux of labeled proteins in the CSF through the glymphatic system (Iliff et al., 2012; Ma et al., 2017). From P5 (*n*^control^=2, *n^prh/prh^=*1), when enlargement of the lateral ventricles was first observed by MRI in *Ccdc39^prh/prh^* rats (Fig. 2B,D-G), to P12-P13 (*n*^control^=3, *n^prh/prh^*=3), after the onset of severe hydrocephalus in this model (Fig. 2C,H-M), mutants exhibit weakened Evans Blue staining of the perivascular spaces around the ventral and lateral surfaces of the middle cerebral artery (MCA) compared with age-matched controls (*P<*0.001; Fig. 7A-D,I,J). At later stages of hydrocephalus, such as P27 (*n*^control^=1, *n^prh/prh^*=1), *Ccdc39^prh/prh^* rats exhibit no staining of the perivascular spaces around the ventral or lateral surfaces of the MCA compared with controls (*P<*0.001) (Fig. 7E-J). Nonlinear regression analysis of the length of tracer staining demonstrated a progressive decline in staining of the perivascular spaces surrounding the MCA of *Ccdc39^prh/prh^* mutants as the hydrocephalus phenotype progressed from P5 to P27 (*P<*0.001) (Fig. 7I,J). Based on this data, we conclude that a progressive impairment in glymphatic-mediated CSF circulation, as traced by Evans Blue dye, exists in this model as *Ccdc39^prh/prh^* mutants develop progressive hydrocephalus and enlargement of the lateral ventricles over the first 4 weeks of life.

## DISCUSSION

The accessibility and affordability of CRISPR/Cas9 genome engineering have allowed multiple scientific groups to generate novel models of congenital hydrocephalus in a variety of organisms, including mice (Morimoto et al., 2019), rats (Emmert et al., 2019) and frogs (Date et al., 2019). The current study demonstrates our successful generation of a robust rat model of neonatal hydrocephalus via the use of CRISPR/Cas9 genome editing to introduce a homozygous splice-site mutation, *Ccdc39^c.916+2T>A^*, in the *Ccdc39* gene that we previously identified in the *prh* mouse mutant.

Using the *L1cam* mutant rat allele in a rat model of XLH, which we previously generated, we demonstrate potential genetic interactions between the *L1cam* and *Ccdc39* genes with phenotypic similarity. Epistatic influences on the expression of genes implicated in the pathogenesis of hydrocephalus have long been of interest to researchers attempting to explain the variable presentation of this condition in laboratory models as well as humans. For example, the phenotype of *L1cam* knockout mice, which includes cognitive deficits, hypoplasia of the corpus callosum, and ventricular enlargement, varies greatly depending on the genetic background of the model (Cohen et al., 1998; Dahme et al., 1997; Itoh et al., 2004; Rolf et al., 2001; Rünker et al., 2003). Patients with *L1CAM* gene mutations present with several conditions that vary considerably both between and within families, suggesting that the penetrance of L1 gene mutations is dependent upon the epistatic influences of other genes (Weller and Gärtner, 2001). We found that rats containing mutations in both genes (*L1cam^y/−^;Ccdc39^prh/prh^*) are smaller, develop earlier and more severe ventriculomegaly and die earlier than either *L1cam^y/−^* or *Ccdc39^prh/prh^* rat mutants. Due to the essential functions of the L1CAM protein in neuronal migration, axon growth and guidance and synaptic plasticity (Itoh and Fushiki, 2015), the disrupted neurogenesis that occurs as a result of the loss of functional L1CAM may exacerbate the impaired motile cilia function and CSF flow necessary for neural cell growth and differentiation in this rat model. Indeed, we found that the lack of motile cilia results in delayed maturation of cerebral cortical neurons, impaired myelination and accelerated neural cell death in this model (Fig. 6). The neurogenesis and neuronal developmental origins of hydrocephalus also have been suggested by a recent study that found multiple neural stem cell fate gene mutations in the largest exome sequencing cohort in human congenital hydrocephalus (Furey et al., 2018a). Further study of neocortical neural development in *L1cam*;*prh* double-mutant rats may clarify the molecular mechanisms of epistasis in hydrocephalus, leading to a better understanding of the heterogeneous presentations of this condition in both rodents and humans. Likewise, future experiments should aim to elucidate the epistatic influences of hydrocephalus-causing gene mutations on the cognitive development of the hydrocephalic brain, as the *L1cam;Ccdc39* double-mutation model is amenable to a variety of behavioral and nonbehavioral assays that were not performed in the current study.

We found that the extent of interaction between *L1cam* and *Ccdc39* is not significant in the double-heterozygote condition. Our data on survival, growth and ventricular volume between *L1cam^y/−^;Ccdc39^wt/prh^* and *L1cam^y/−^* rats as well as *L1cam^wt/−^;Ccdc39^wt/prh^* and *L1cam^wt/−^* rats suggest that the heterozygous level of CCDC39 or L1CAM protein expression is sufficient to maintain ependymal cilia beating or neuronal cell growth, respectively, and does not affect the XLH phenotype or primary ciliary dyskinesia phenotype. As such, our rat genetics data exclude the human heterozygous *CCDC39* mutant allele and heterozygous *L1CAM* allele from the candidate genetic modifier elements that alter the disease severity of XLH and primary ciliary dyskinesia.

In previous experiments using the hydrocephalus rodent model, a variety of inflammatory responses were observed, such as astrocytic activation in hyh (hydrocephalus with hop gait) mutant mice (Roales-Buján et al., 2012) and reactive microgliosis in hydrocephalic H-Tx rats, which can eventually inhibit neurite outgrowth and brain recovery (Mangano et al., 1998; Miller and McAllister, 2007). In kaolin-induced hydrocephalus rats, altered inflammatory gene expression due to hydrocephalus was documented (Deren et al., 2010) in addition to the reactive astrocytes and microglial response (Deren et al., 2010; Khan et al., 2006), although there has been criticism that kaolin itself could produce a global inflammatory responses in the brain (Orešković and Klarica, 2011). In a mouse model of posthemorrhagic hydrocephalus, Toll-like receptor 4–nuclear factor kB (NF-kB) signaling has been described as an important pathway in mediating hydrocephalus development by causing further damage to the ependymal cells or CSF hypersecretion from the choroid plexus epithelium (Karimy et al., 2017; Simard et al., 2011). The activation of NF-kB is also known to interfere with ependymal ciliogenesis (Lattke et al., 2012). However, little is known about the inflammatory molecular pathway that mediates and can exacerbate hydrocephalus development from a relatively early phase in a model with no preceding hemorrhage. In this study of neonatal hydrocephalus caused by dysfunction of motile cilia in the ependyma and choroid plexus, and thus without pre-existing hemorrhage, we have shown that inflammatory macrophage migration occurs at the early stage of ventricular expansion (P5) and that pro-inflammatory cytokine expression, as well as further immune cells migration, follows in the middle to later disease stages (P11-P30) mainly around the subarachnoid/perivascular space as well as the periventricular area in our model. To elucidate subtypes of pro-inflammatory signals recruiting macrophages to the brain, we evaluated MCP1 expression in our rat model of neonatal hydrocephalus. We found significantly increased MCP1 signals at P11 that were expressed in neurons and endothelial cells, as well as macrophage invasion starting from P5 with relatively mild ventriculomegaly. Increased MCP1 expression has been commonly reported in the CSF of patients with posthemorrhagic (Killer et al., 2010) or idiopathic normal pressure hydrocephalus (Pfanner et al., 2018). Our data suggest that, in neonatal hydrocephalus, MCP1 is one of the key molecules that recruits the initial leukocyte infiltration and accelerates neuroinflammation with macrophage and neutrophil extravasations. Future investigations in this model will elucidate whether MCP1 inhibitors can ameliorate neuroinflammation and, potentially, hydrocephalus progression or functional outcomes, as shown in other disease models such as experimental autoimmune encephalomyelitis (Ge et al., 2012) or ethanol-induced neurodegeneration (Zhang et al., 2018).

Subarachnoid/subpial hemorrhage was observed at P11, followed later by hemorrhage into the lateral ventricles, possibly as a result of inflammatory insult or distention of the neural tissues, which eventually ruptured the vessels. MMP9 activation in astrocytes was also seen in the neocortical area, with arachnoid/pial membrane dissociation from the parenchymal tissue with accumulated fluid and occasional bleeding. MMP9 in astrocytes has been reported to be induced by many pro-inflammatory cytokines such as tumor necrosis factor-α, interleukin-1β or Toll-like receptor 2 along with reactive oxygen species in the setting of CNS inflammation (Min et al., 2015; Rosenberg, 2002; Yang et al., 2015). MMP9 is known to degrade collagen IV and collagen V, leading to tissue destruction and eventually blood–brain barrier disruption (Könnecke and Bechmann, 2013); therefore, it could exacerbate pathogenesis of the hydrocephalus by inducing bleeding and more inflammatory cell migration.

Although it is challenging to differentiate pia mater from the subarachnoid membrane by histology, some fluid accumulation in *Ccdc39^prh/prh^* mutant rats was seen under the layer containing leptomeningeal vessels over the brain surface (arrows in Fig. 4A), which implies that fluid accumulated under pia mater. Pia mater lacks tight junctions and is known to be permeable to water (Alcolado et al., 1988). Thus, it is possible that water comes across the pia mater as a result of over-accumulation of CSF in the subarachnoid space in our model of neonatal hydrocephalus. Because CD68-positive macrophage invasion into the periventricular region was seen in P5 mutants before the subarachnoid hemorrhage started, the inflammatory reaction is primarily a result of the hydrocephalus insult. Conversely, this eventual subarachnoid hemorrhage and exacerbated inflammatory cell recruitment in the subarachnoid space could have affected and further impaired the glymphatic flow along perivascular spaces by hampering the absorption of CSF.

An increased number of cells positive for cell death signal (TUNEL) were observed in the periventricular region in our *Ccdc39^prh/prh^* pups at P1-P5. This distribution was very similar to that of CD68-positive macrophages at this age. Because there was no significant difference in the number of cleaved caspase-3-positive cells to label apoptotic cells at P11 in our model (data not shown), it is likely that significant cell death in the periventricular region occurs at an earlier phase (P1-P5 in our model) rather than at the later stage of the disease in our model. Del Bigio and colleagues reported that although there were significantly more overall TUNEL-positive cells in kaolin-induced hydrocephalus rats 3-4 weeks after kaolin injection into the cisterna magna compared with controls, most of the TUNEL-positive cells (more than 95%) morphologically looked like non-neuronal cells (Del Bigio and Zhang, 1998). In the evaluation of axonal damage in kaolin-induced hydrocephalus rats, Ding et al. found that there was little evidence for neuronal cell death at any stage of hydrocephalus, irrespective of the severity of axonal degeneration (Ding et al., 2001). Therefore, although periventricular axons are primarily damaged by ventriculomegaly, neurons rarely die initially until the periventricular white matter is completely eroded (Del Bigio, 2010) or thalamic retrograde axonal degeneration leads to cell apoptosis (Mori et al., 2002). The TUNEL-positive cells in the periventricular region in our *Ccdc39^prh/prh^* rats may have been glia or other non-neuronal cells, as suggested in a previous report (Del Bigio and Zhang, 1998), although more investigation is needed.

Impaired myelination and poorly developed neurofilaments at P11 were also observed in this study. Although myelination had barely started at P5, even in control rats, and there was no significant difference in the number of mature oligodendrocytes in *Ccdc39^prh/prh^* mutant and control rats (Fig. S2A), there was a tendency for decreased neurofilament density in *Ccdc39^prh/prh^* mutants at P5 (Fig. S2B). Both periventricular axons and the myelin sheath are reported to be the major sites of injury in the hydrocephalic brain due to tissue extension, hypoxic insult (Del Bigio, 2001, 2010) or inflammatory insult, as observed in our hydrocephalus model. Oligodendrocytes are reported to be vulnerable to the hydrocephalus insult at young ages, such as up to P35 for ferrets (Di Curzio et al., 2013), and the impairment of myelination has been described in neonatal hydrocephalus both in human (Hanlo et al., 1997) and animal models (Del Bigio et al., 1997, 2003). In 3-week-old rats with hydrocephalus induced by kaolin injection, the myelin sheath around axons greater than 0.4 μm in diameter became significantly thinner 1 week after injection, followed by irreversible axon loss if left untreated (Del Bigio et al., 1997). Whether dysmyelination precedes axonal loss or vice-versa remains controversial, but our result at P5 implies that axonal immaturity precedes delayed myelination or demyelination. Brain gene expression in hydrocephalic rats related to synaptogenesis, myelination, cell cycles and other important signaling seems to be altered, depending on age (Balasubramaniam and Del Bigio, 2002). Therefore, our result might only apply to the sequelae of a specific age of the hydrocephalus development in which ventriculomegaly starts from P5 in rats.

The glymphatic system of humans and rodents serves as a CSF conduit from the subarachnoid space through periarterial spaces into the brain parenchyma for drainage of parenchymal waste and solutes through perivenous spaces into meningeal and cervical lymphatic vessels (Klebe et al., 2019; Rasmussen et al., 2018). Impaired glymphatic-mediated fluid exchange has been associated with multiple neurological disorders, including Alzheimer's disease (Iliff et al., 2012), traumatic brain injury (Iliff et al., 2014) and stroke (Gaberel et al., 2014). The glymphatic system is hypothesized to play a role in dementia experienced by patients with normal pressure hydrocephalus (Rasmussen et al., 2018; Ringstad et al., 2017) as well as inflammation and motor and cognitive defects in mice undergoing craniectomy (Plog et al., 2019). In concordance with the recent discovery of the perinatal pattern of development of the glymphatic system (Munk et al., 2019), we found impaired circulation of CSF tracers along the glymphatic system in our neonatal hydrocephalus model, which constitutes the first report of the glymphatic system's involvement in neonatal hydrocephalus. Specifically, we found that *Ccdc39^prh/prh^* mutant rats show reduced uptake of Evans Blue tracing dye along the perivascular spaces of the ventral and lateral surfaces of the middle cerebral artery as the progressive hydrocephalus phenotype becomes more severe from P5 to P27. Given the importance of CSF pressure gradients between the subarachnoid space and venous sinuses in driving CSF outflow (Klebe et al., 2019), we believe that reduced perivascular fluid bulk movement, which could be explained by altered CSF hydrodynamics or intracranial pressure in this model, causes reduced glymphatic uptake of CSF in *Ccdc39^prh/prh^* rats. Alternatively, it is also possible that the lack of motile cilia-dependent CSF flow affects the structural development and maturation of this system. Due to the crucial solute-clearance role of the glymphatic system, its reduced function in our model of neonatal hydrocephalus may partly explain the cognitive impairment of pediatric hydrocephalus patients if left untreated (Mangano et al., 2016).

Although understanding of the organization and function of the glymphatic pathway has quickly advanced since its original identification in the rodent brain in 2012 (Iliff et al., 2012), mechanisms of glymphatic dysfunction in conditions such as hydrocephalus are predominantly theoretical (Klebe et al., 2019; Rasmussen et al., 2018). In line with emerging information that the mouse glymphatic system begins developing in the hippocampus at P1 and is fully established in the cortex by 2 weeks of age (Munk et al., 2019), future studies using shunt treatments such as the ventriculo-subcutaneous shunt (Harris et al., 1994, 1996; Jones et al., 1995) in the *Ccdc39^prh/prh^* rat model can address whether neonatal hydrocephalus impairs normal development of the glymphatic system. Therefore, we present the *Ccdc39^prh/prh^* rat as a strong model for studying the molecular basis of impaired neocorticogenesis, glymphatic fluid exchange and neurocognitive and motor skill development in neonatal hydrocephalus.

A primary limitation of the study was the small sample size of *L1cam^wt/−^;Ccdc39^prh/prh^* mutants used in ventricular volume analysis at P5 (*n*=2) and *Ccdc39^prh/prh^* mutants used in histological analysis of MCP1 (*n*=3), MBP (*n*=3), NF-H (*n*=3) and cell death (*n*=3) at P1-P11. The primary challenge of generating a new transgenic *Ccdc39^prh/prh^* rat model of hydrocephalus and interbreeding it with a pre-existing transgenic *L1cam^y/−^* rat model of XLH is obtaining and maintaining mutants at ages ranging from P1 to P90, which is confounded by the unpredictable death of rodents with hydrocephalus (Di Curzio, 2018). Although our findings of ventricular enlargement, reduced survival and growth, increased MCP1 expression, higher number of TUNEL-positive cells, downregulation of MBP and NF-H, and reduced Evans Blue dye uptake into the glymphatic system in the *Ccdc39^prh/prh^* mutant rat demonstrate statistically significant differences between groups, the *post-hoc* power analysis of these studies demonstrates statistical power ranging from 62.1% to 100%. Future experiments of ventricular dilation and expression of neuroinflammatory markers in the early hydrocephalic brain in this model will further elucidate the pathogenesis of hydrocephalus in *Ccdc39^prh/prh^* mutants in the absence and presence of other genetic modifiers.

In comparison to *prh* mutant mice (Abdelhamed et al., 2018), *Ccdc39^prh/prh^* mutant rats similarly exhibited loss of brain CCDC39, decreased growth and survival throughout the weaning phase, and progressive hydrocephalus with dilation of the lateral ventricles from P5 that progressed into severe hydrocephalus by P11. *Ccdc39^prh/prh^* mutant rats showed slightly slower hydrocephalus development than *prh* mutant mice, as hydrocephalus in *Ccdc39^prh/prh^* mice progressed from P1 to a severe form by P7. We found that *Ccdc39^prh/prh^* rats developed dome-shaped heads, which indicates that the unfused skull suture might alleviate the elevation in intracranial pressure (ICP) at this neonatal stage (Jones et al., 2000), although ICP is expected to increase once the skull sutures fuse between P12 and P20 (Grova et al., 2012; Roth et al., 1997) and the ventriculomegaly reaches moderate proportions (Jones et al., 2000). Although the *prh* mutant mouse was useful for identifying the direct defects of motile cilia (Abdelhamed et al., 2018), the small anatomical size of the murine model constrains its use for surgical procedures such as ICP recording (Hiploylee and Colbourne, 2014) and ventricular–subcutaneous shunting (Santos et al., 2016) that can more easily and accurately be performed in rats. Ultimately, the use of these advanced surgical procedures in our larger rodent model of neonatal hydrocephalus can provide new insights into the role of intracranial pressure change in neonatal hydrocephalus.

In conclusion, we report our successful generation of a novel rat model of neonatal hydrocephalus using CRISPR/Cas9 to introduce a recessive splice donor site mutation into the rat *Ccdc39* gene. *Ccdc39^prh/prh^* rat mutants recapitulate the progressive hydrocephalus and decreased growth and survival of *prh* mice. We further found evidence for a novel genetic interaction between two gene mutations in *L1cam* and *Ccdc39* in neonatal hydrocephalus development, which supports their potentially common physiological roles in normal brain development, such as in neuronal growth, survival and maturation that occur downstream of ependymal cilia-mediated CSF flow. Inflammatory reactions via impeded CSF flow as demonstrated in our model suggest the active rather than passive roles of CSF flow retardation in altering neocortical maturation, which can cause further brain damage in neonatal hydrocephalus. Impaired glymphatic-mediated CSF circulation in our model provides new insights into the glymphatic system, which may be involved in normal brain development and the pathogenesis of neonatal hydrocephalus. Future experiments should investigate mechanisms of the inflammatory response as well as those regulating glymphatic flow and clearance using a combination of molecular techniques and advanced imaging and surgery. Ultimately, improved understanding of CSF exchange mechanisms under conditions of inflammation could lead to new therapeutic strategies for hydrocephalus by altering CSF circulation.

## MATERIALS AND METHODS

### CRISPR/Cas9-based rat *Ccdc39* mutant generation

CRISPR/Cas9-based genome editing was performed as described previously (Emmert et al., 2019) with the following modifications: Single guide (sg)RNAs (42 ng/µl of each) were mixed with 200 ng/µl Cas9 protein (Thermo Fisher Scientific), and single-stranded (ss)DNA donor oligonucleotides (oligos) containing the *Ccdc39^c.916+2T^*^>*A*^ mutation and engineered *Hin*dIII restriction site (AAGCTT) were incubated at 37°C for 15 min to form a ribonucleoprotein complex. The CRISPR/Cas9 complex mixture and donor oligos were injected into the cytoplasm of single-cell stage embryos (*n*=38) of Sprague Dawley rats using a piezo-driven microinjection technique. Following embryo transfer into the oviducal ampulla of pseudopregnant females, a founder generation (F0) of rats (*n*=26) was born and Sanger sequenced. F1 mutants were obtained through backcrossing of F0 mosaic animals to wild-type Sprague Dawley rat. All following generations of offspring were genotyped using a TaqMan Sample-to-SNP probe-based assay with custom probes for the rat *Ccdc39^c.916+2T>A^* mutation (assay ID #AN322UG for *Ccdc39^prh^*). Breeding with and genotyping of the rat *L1cam* mutant allele were performed as described previously (Emmert et al., 2019). Rats analyzed using MRI, histology and glymphatic-mediated CSF tracing were of F2 or F3 generations. Only male rats homozygous for *L1cam* mutation (*L1cam^y/−^*, *L1cam^y/−^;Ccdc39^wt/prh^*, *L1cam^y/−^;Ccdc39^prh/prh^*) were included because female homozygous *L1cam* mutants cannot be obtained with our breeding techniques due to sterility of male *L1cam* mutants with XLH. Rats were housed in specific pathogen-free conditions and all experiments were performed according to the Institutional Animal Care and Use Committee guidelines of the Cincinnati Children's Hospital Medical Center.

### Western blotting

Western blotting was performed as previously described (Abdelhamed et al., 2018; Emmert et al., 2019). Briefly, whole P11 rat brain lysates in RIPA buffer [50 mM Tris-Cl pH 7.4, 150 mM NaCl, 5 mM EDTA, 1% Nonidet P-40, 1% sodium deoxycholate, 0.1% SDS, 1% proteinase inhibitor cocktail (Thermo Fisher Scientific)] were separated, transferred to a PVDF membrane and probed with anti-CCDC39 (1:1000, #HPA035364, Sigma-Aldrich), anti-β-tubulin (1/1000, #T8660, Sigma-Aldrich), and anti-rabbit/mouse IgG-IRDye680RD/800CW (LI-COR) antibodies. Fluorescent signals were detected using the Odyssey Imaging System (LI-COR).

### Magnetic resonance imaging

MRI data were acquired on a Bruker 7T Avance horizontal bore small animal MRI scanner (Bruker, Billerica, MA). Control and mutant rats ranging in age from P1 to P90 were scanned. Rat pups at P1-P7 were anesthetized using hypothermia because isoflurane is not effective at this age. Rats at P11 and older were anesthetized with 2.5–3.5% isoflurane in air, positioned supine and scanned. Rat pups at P20 and older were secured on the MRI animal bed with their teeth on a bite bar. For animals under isoflurane anesthesia, respiration was monitored and body temperature was maintained at 36°C–38°C using an animal monitoring system (SA Instruments, Stony Brook, NJ). Animals were positioned in the coil and centered in the bore of the magnet. Fluid-sensitive images were acquired with a fat-saturated 3D T2 RARE sequence (Hennig et al., 1986) using the following parameters: repetition time 2 s, echo time 264 ms, echo spacing 11 ms, RARE factor 60, receiver bandwidth 104 kHz, averages 2, matrix 320×108×96, field of view 48×16×144 mm and total scan time 4 min 40 s. DICOM images of controls and mutants were imported into the ImageJ package Fiji. Volumes (mm^3^) of the lateral ventricles, third ventricle, fourth ventricle and pineal recess were measured using the Surfaces feature of the Imaris software (Bitplane Scientific Software) (voxel size: *x*=0.150 mm, *y*=0.148 mm, *z*=0.150 mm).

### Tracing of glymphatic-mediated CSF flux

The pattern of CSF flow along the glymphatic system was assessed as described previously (Iliff et al., 2012). Briefly, P5-P27 rats were deeply anesthetized using ketamine (100 mg/kg) and xylazine (10 mg/kg). About 2-10 μl of 4% Evans Blue dye in PBS (0.96 kDa; Sigma-Aldrich) was injected into the cisterna magna at a rate of 1-2 μl/min with a 33 G needle connected to the micropump 11 elite (Harvard Apparatus). To measure the uptake of Evans Blue dye into the brain over 60 min, the rats were sutured and administered carprofen according to the survival surgery guidelines of Cincinnati Children's Hospital Medical Center. Rats were perfusion fixed for 60 min after the injection. Brains were fixed in 4% PFA overnight and imaged using a stereomicroscope. The length (mm) of Evans Blue staining of the ventral and lateral surfaces of the middle cerebral artery of controls and *Ccdc39^prh/prh^* mutants was measured using the freehand line tool on Fiji.

### Histology, immunohistochemical/immunofluorescence staining and cell quantification

Brains in the skull removed from *Ccdc39^prh/prh^* and control rats aged P1-P30 (*n*=28) were fixed in formalin for 24 h without systemic perfusion and embedded in paraffin after decalcification for two overnights (Shandon TBD-2, Thermo Fisher Scientific) and ethanol dehydration. Microtome sections (5 µm thick) after deparaffinization and rehydration were used in H&E staining or immunohistochemical staining following antigen retrieval in citrate buffer (pH 6) for 45 min. For the immunohistochemical/immunofluorescent staining, sections were incubated with primary antibodies of either anti-rabbit MMP9 (N-terminus) (1:100; Proteintech, 10375-2AP), anti-mouse GFAP (1:500; Sigma-Aldrich, G3893), anti-mouse CD68 (1:100; Abcam, ab31630), anti-rabbit MPO (1:50; Abcam, ab9535), anti-rabbit MCP1 (1:100; Bioss, BS1101R), anti-mouse NeuN (1:1000; Chemicon, MAB377B), anti-rabbit MBP (1:500; Abcam, ab40390) or anti-mouse NF-H (1:500; BioLegend, 801701) overnight after blocking in 10% normal donkey serum and 0.1% Triton X-100 in PBS for 1 h. After stringent washing and subsequent incubation with fluorophore- or horseradish peroxidase-conjugated secondary antibodies and isolectin GS-IB_4_, the Alexa Fluor 594 Conjugate (IB4) (Thermo Fisher Scientific) was incubated along with secondary antibodies to label endothelial cells. Sections were counterstained with DAPI (Sigma-Aldrich) or hematoxylin (Vector Laboratories), respectively. The terminal deoxynucleotidyl transferase dUTP nick end labeling (TUNEL) assay was performed according to the manufacture's protocol using Apop Tag Fluorescein *In Situ* Apoptosis Detection Kit (Millipore).

Fluorescently stained sections were observed under a confocal laser scanning microscope (Nikon A1R Ti-E inverted microscope). ImageJ software (NIH) was used to estimate the MBP-positive (P11) and NF-H-positive (P5, P11) areas out of the total brain area in a section, as well as MBP-positive cells per area at P5 in comparable sections. To quantify TUNEL-positive cells relative to the number of DAPI-positive nuclei, comparable sections were selected to show periventricular regions. The number of TUNEL-positive cells was manually counted and divided by the number of nuclei counted using ImageJ software. The MCP1 immunohistochemistry signal reacted with DAB substrate at the surface of the brain cortex, deep parenchyma and around the lateral ventricles was observed using a Nikon A1R Ti-E inverted microscope with a 40× objective lens for P11 and an Olympus DP71 microscope with 40× objective lens for P5 evaluations. Signal intensity was analyzed using ImageJ software.

### Statistical analysis

All values are expressed as the mean±standard deviation (s.d.) or standard error of the mean (s.e.m.), as indicated. Justification for the use of parametric two-tailed statistical analyses, including two-way analysis of variance (ANOVA), Kaplan–Meier survival analysis and the Student's *t*-test, is derived from normality testing of the data using Shapiro–Wilk tests. Statistical computation of group differences among more than two groups was performed with two-way ANOVA with Holm–Šidák control for multiple comparisons. Survival data (defined as the number of days until death) were analyzed using the log-rank procedure of Kaplan–Meier survival analysis. Body weights and length of Evans Blue staining along the ventral and lateral surfaces of the middle cerebral artery were analyzed by comparing fits of nonlinear regression curves and best-fit values. The differences between the two groups were compared using the Student's *t*-test. *P*<0.05 was considered statistically significant. All statistical computations were performed in GraphPad Prism.

## Supplementary Material

Supplementary information
