# Supplementary material for: Impaired neural differentiation and glymphatic CSF flow in the Ccdc39 rat model of neonatal hydrocephalus: genetic interaction with L1cam
Source: Dis Model Mech. 2019 Nov 21;12(11):dmm040972. doi: 10.1242/dmm.040972 (PMC6898999; doi:10.1242/dmm.040972)
Supplement: Supplementary information [file dmm-12-040972-s1.pdf]

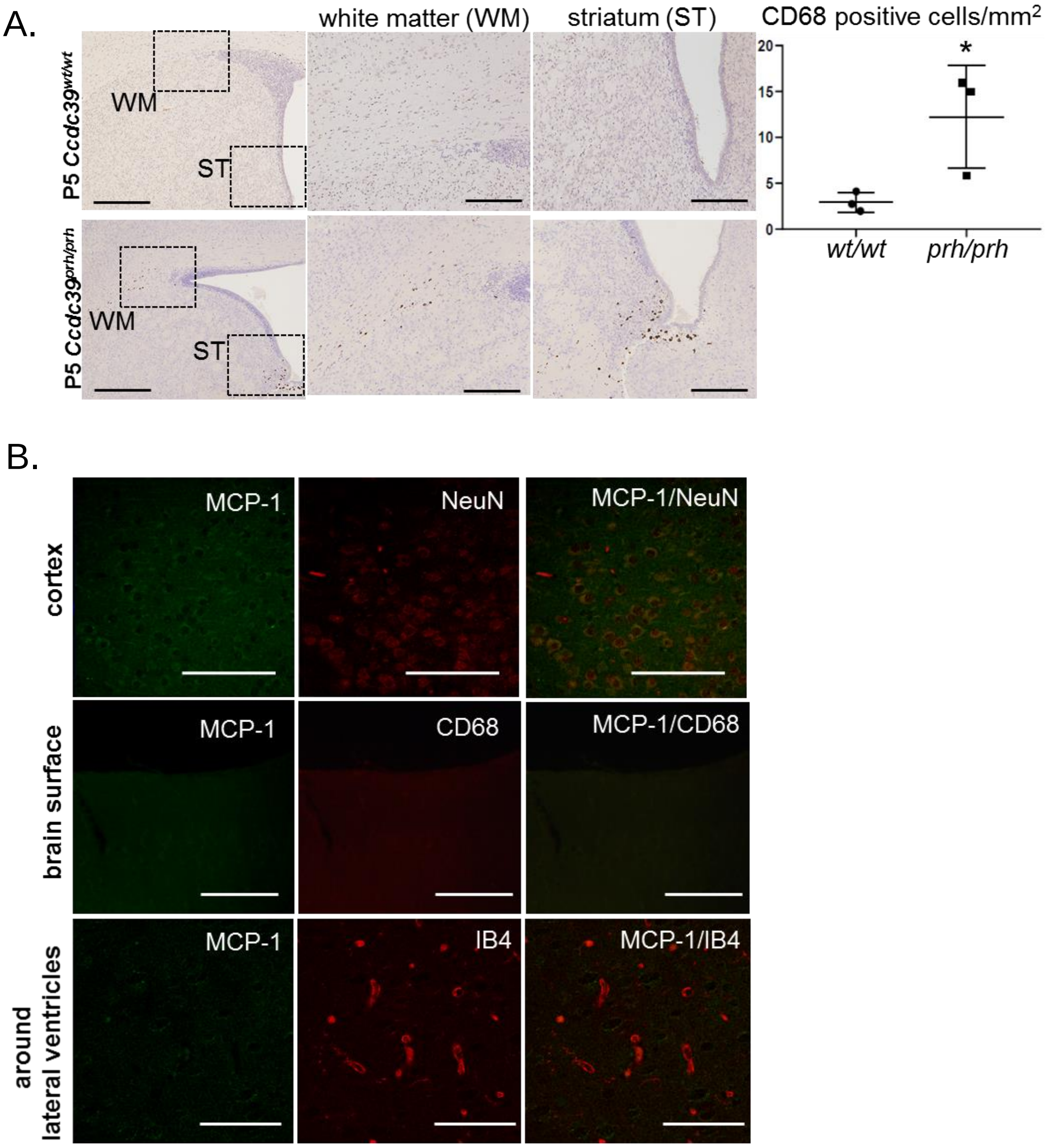

Figure S1. “Immune cell infiltration in *Ccdc39*$^{prh/prh}$ brain”

(A) CD68 staining in *Ccdc39*$^{wt/wt}$ and *Ccdc39*$^{prh/prh}$ at P5. More number of CD68 positive macrophages are seen in white matter and striatum in *Ccdc39*$^{prh/prh}$ mutant rats compared to wild type rats at P5 (scale bars = 500, 200, 200 μm from left to right, mean ± SD, p = 0.046, n = 3 in each group) (WM = white matter, ST = striatum).

(B) MCP-1 expressions in P11 *Ccdc39*$^{wt/wt}$*.* Double staining of Monocyte chemoattractant protein (MCP) -1 with either NeuN, CD68, or Isolectin B4 (IB4) shows that in wild type rats, MCP-1 is weakly expressed in NeuN-positive neurons. In wild type rats, CD68 positive macrophages are not migrated on the surface of the brain, and therefore MCP-1 is observed on brain surface. MCP-1 is barely expressed in endothelial cells labeled with IB4 in deep brain parenchyma (scale bars = 100, 100, 50 μm from top to bottom).

Disease Models & Mechanisms • Supplementary information

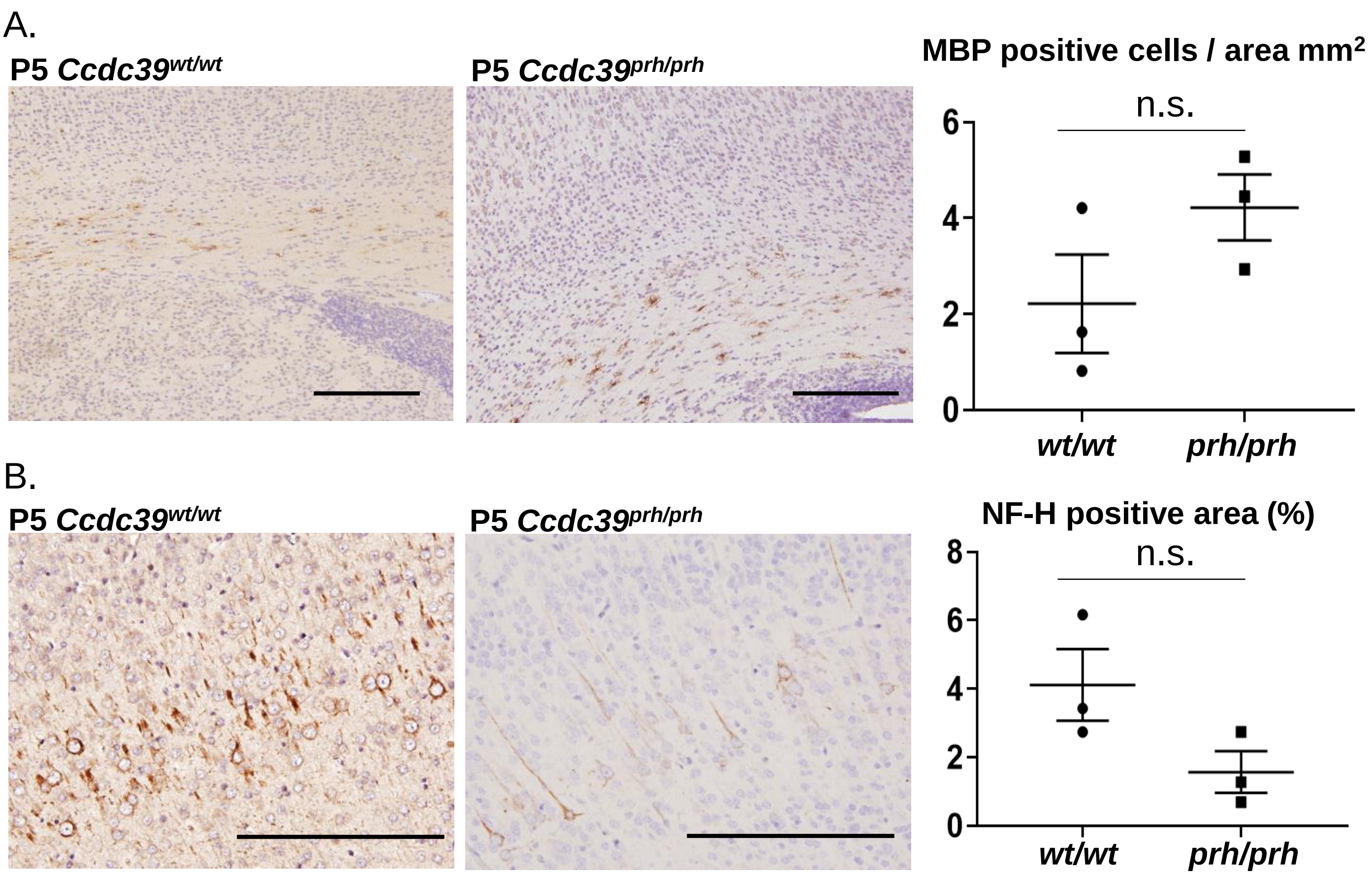

Figure S2 “Neural cell differentiation in *Ccdc39*$^{prh/prh}$ rats”

(A) Myelination in *Ccdc39*$^{wt/wt}$ and *Ccdc39*$^{prh/prh}$ at P5. At P5, myelination as seen at P11 is not yet started even in wild type rats. When quantifying the number of Myelin basic protein (MBP)-positive mature oligodendrocytes, the number of mature oligodendrocytes in *Ccdc39*$^{prh/prh}$ mutant rats was comparable to wild type rats (scale bars = 200 μm, mean ± SD, $p = 0.18$, n = 3 in each group).

(B) Neurofilament in *Ccdc39*$^{wt/wt}$ and *Ccdc39*$^{prh/prh}$ at P5. There was a tendency of reduced neurofilament formation stained with neurofilament-H subunit (NF-H) in in *Ccdc39*$^{prh/prh}$ mutant rats at P5 compared to wild types, although not statistically significant (scale bars = 200 μm, mean ± SD, $p = 0.11$, n = 3 in each group).
